# Common Gene Modules Identified for Chicken Adiposity by Network Construction and Comparison

**DOI:** 10.3389/fgene.2020.00537

**Published:** 2020-05-29

**Authors:** Zhuoran Gao, Ran Ding, Xiangyun Zhai, Yuhao Wang, Yaofeng Chen, Cai-Xia Yang, Zhi-Qiang Du

**Affiliations:** ^1^College of Animal Science, Yangtze University, Jingzhou, China; ^2^College of Animal Science and Technology, Northeast Agricultural University, Harbin, China

**Keywords:** chicken, WGCNA, gene network, modules, fat deposition

## Abstract

Excessive fat deposition can cause chicken health problem, and affect production efficiency by causing great economic losses to the industry. However, the molecular underpinnings of the complex adiposity trait remain elusive. In the current study, we constructed and compared the gene co-expression networks on four transcriptome profiling datasets, from two chicken lines under divergent selection for abdominal fat contents, in an attempt to dissect network compositions underlying adipose tissue growth and development. After functional enrichment analysis, nine network modules important to adipogenesis were discovered to be involved in lipid metabolism, PPAR and insulin signaling pathways, and contained hub genes related to adipogenesis, cell cycle, inflammation, and protein synthesis. Moreover, after additional functional annotation and network module comparisons, common sub-modules of similar functionality for chicken fat deposition were identified for different chicken lines, apart from modules specific to each chicken line. We further validated the lysosome pathway, and found *TFEB* and its downstream target genes showed similar expression patterns along with chicken preadipocyte differentiation. Our findings could provide novel insights into the genetic basis of complex adiposity traits, as well as human obesity and related metabolic diseases.

## Introduction

The global obesity pandemic and related metabolic syndromes are currently devastating the human society, by threatening human health and decreasing life expectancy (Blüher, 2019). To find effective therapy for metabolic diseases, current research efforts employ large-scale genomics and systems biology methods, to understand better the biology and physiology of adipose tissues (Dyar et al., 2018; Guan et al., 2018; Pellegrinelli et al., 2018; Zhao et al., 2018; Brial et al., 2019; Justice et al., 2019; van der Klaauw et al., 2019). However, adipose tissues are of complex and heterogeneous origins, such as depot-specific (subcutaneous, abdominal, inguinal, etc.), different tissue-types (white, brown, and beige), and composition of different cell types (immune cells, preadipocytes, stromal cells, neurons, etc.) (Lee et al., 2014; Jeffery et al., 2016; Ghaben and Scherer, 2019; Luong et al., 2019; Sebo and Rodeheffer, 2019). It’s rather difficult to disentangle the molecular circuits driving the growth and development of adipose tissue (Tang and Lane, 2012; Ahrends et al., 2014; Cohen and Spiegelman, 2016).

Gene network approach, as one method of integrative analysis on high-throughput transcriptome profiling and a variety of other omics data, helps discover successfully the structural and functional gene modules and molecular signaling pathways for human diseases (Barabási and Oltvai, 2004). Different statistical and machine learning methods were developed and refined for the construction of gene networks, e.g., the gene coexpression network (Langfelder and Horvath, 2008; Ranola et al., 2013), and the Bayesian network (Chen et al., 2008; Yang et al., 2009). Later, integrative genomics methods are effective in combining different omics data (e.g., transcripomics, proteomics, metabolomics), to pinpoint key biochemical and molecular biomarkers (Lee et al., 2016; Emilsson et al., 2018; Ghaemi et al., 2019). In farm animals, the gene-coexpression network were recently used to study cattle reproduction, feed intake, meat quality, immune response, and functional annotation of gene functions (Fortes et al., 2010; Beiki et al., 2016, 2018; Buchanan et al., 2016; Mateescu et al., 2017; de Oliveira et al., 2018; Gonçalves et al., 2018; Alexander et al., 2019). In chickens, network methods were also employed on the investigation of growth and reproduction traits (Zhang et al., 2017; Wang et al., 2018).

Intensive selection on growth rate and feed efficiency traits in the past several decades makes the broiler industry one of the most efficient animal production systems. However, fast growth could bring along excessive fat deposition, and cause economic loss and processing burden to the broiler industry. To breed broiler lines with less fat, recent efforts are focused on understanding the molecular genetics of adipose tissue growth and development in the chicken (Siegel, 2014). Broiler lines were divergently selected for abdominal fat content (Guo et al., 2011; Baéza and Le Bihan-Duval, 2013), and a systematic approach integrating genetic, genomic, cellular, and molecular studies were performed, to discover important genes and molecular pathways involved in adipogenesis (Wang et al., 2007; Zhang et al., 2017; Cui et al., 2018; Na et al., 2018; Wang et al., 2019). Similarly, other chicken lines continuously under divergent selection for abdominal fat content (French lines) or body weights (Virginia lines) were also constructed, to study the molecular genetics of fat deposition or growth rate (Baéza and Le Bihan-Duval, 2013; Siegel, 2014). However, to our knowledge, analysis on gene network construction and functional comparison on chicken adipogenesis is very limited.

In the present study, we constructed gene co-expression networks for four different transcriptome profiling datasets collected on abdominal fat tissues or isolated preadipocytes from chicken lines under divergent selection for adiposity, and compared the structural characteristics of the obtained network modules, to see if common molecular programs exist for adipogenesis. We identified important gene modules for adipogenesis, and interestingly, discovered common gene modules shared by different chicken lines, too. Our results provide evidences that even though genetic underpinnings of adiposity in chickens are complex, common molecular features could still exist, which renders novel insights on animal breeding practices, and also genetic investigation on human obesity.

## Materials and Methods

### Ethics Statement

All animal work was conducted in compliance with the recommended guidelines described in the Guide for the Care and Use of Laboratory Animals, and was approved by the Animal Care and Use Committee of Hubei Province, China (YZU-2018-0031).

### Animals

Two chicken lines under divergent selection for abdominal fat content were used in the present study. One chicken line was from the Northeast Agricultural University broiler lines divergently selected for abdominal fat contents (NEAUHLF) since 1996, using abdominal fat percentage (AFP) and plasma very low-density lipoprotein (VLDL) concentration as selection criteria (Guo et al., 2011). These lines were developed from a common base grandsire line, Arbor Acres. Birds were bi-directionally selected for abdominal fat content, whereas bodyweights were kept the same for both lines. Abdominal fat percentages of the fat and lean broiler lines were significantly different from each other since the 4th generation, and detailed description on the selection procedure and housing conditions could be found in the previous report (Guo et al., 2011). The other French chicken line was from fat and lean chickens bred and raised at INRA UE1295 Pôle d’Expérimentation Avicolede Tours, F–37380 Nouzilly, France, as described previously (Resnyk et al., 2013, 2017).

### Transcriptome Datasets

In order to explore the genes and molecular pathways affecting adipose tissue growth and development, we analyzed four sets of transcriptome data in the present study: one from the cellular perspective (*in vitro* preadipocyte differentiation), and the other three from adipose tissue perspectives (datasets downloaded from the public database). At the cellular level, microarray data on the differentiation of abdominal preadipocytes of NEAUHLF were collected. We briefly described the whole procedure as follows. Male birds at 10 days of age from the lean and fat lines at the 13th generation were selected, which were offsprings of the families with the highest and lowest AFP according to their slaughtered sib information, respectively. For each line, abdominal adipose tissues of 15 male birds were excised and pooled together. All pooled abdominal adipose tissues were then digested, filtrated, centrifuged, thus allowed the separation of floating adipocytes from the preadipocytes. The stage when the primary preadipocytes were collected was defined as the 0001 time point. And preadipocytes were passaged once, and harvested when 50% confluent (termed as -12 h) and 95% confluent (termed as 0 h). From the time point 0 h onward, preadipocytes were divided into two groups, the control group, and the group treated with 160 μM oleate. Cultured preadipocytes were divided into four groups, including preadipocytes from the lean and fat lines cultured without oleate treatment (LC and FC), or with oleate (LO and FO), respectively. Then preadipocytes in both groups were cultured continuously for 120 h, and samples were collected at four time points (12, 24, 72, and 120 h). Preadipocytes at each time point were collected, and total RNA was prepared from preadipocytes using Trizol reagent (Invitrogen) following the manufacturer’s instructions. Then, cDNA was prepared by oligo(dT)-primed reverse transcription. Labeled cRNA probes were prepared using an IVT Labeling Kit (Affymetrix, Inc.) according to the manufacturer’s protocol. The cRNA were fragmented, heated, loaded onto the Affymetrix probe array cartridge (Affymetrix, Inc.), and then hybridized, washed, and scanned at 560 nm using a confocal scanner. Raw data sets were normalized with Microarray Suite 5.0 (MAS5) and limma package in the R statistical environment (Ritchie et al., 2015; R Core Team, 2019). The raw microarray data of chicken preadipocyte differentiation were deposited into the National Center for Biotechnology Information (NCBI) Gene Expression Omnibus, and could be accessed through GEO Series (accession number: GSE51330).

At the tissue level, three datasets for abdominal fat tissue were accessed from the public domain, one microarray dataset from NEAUHLF, and one RNA-seq and one microarray dataset from the French chicken line, respectively. For the NEAUHLF dataset, 10 birds of the 8th generation were chosen based on the AFP values (five had the highest AFP and the other five had the lowest), and were slaughtered at 7 weeks. Abdominal fat tissues were collected for RNA extraction, and submitted for the GeneChip Chicken Genome Array (Wang et al., 2007). Data were downloaded through GEO (accession number: GSE8010). For the French lines, RNA-Seq and microarray datasets were collected from abdominal adipose tissues isolated from 7-week-old fat and lean chickens, as described previously (Resnyk et al., 2013, 2017), and were downloaded from the public GEO database (accession numbers: GSE42980 and GSE37585), respectively.

### Construction of Weighted Gene Co-expression Network and Identification of Significant Modules

To identify gene co-expression network modules associated with preadipocyte differentiation and adipose tissue growth and development in chickens, the weighted gene co-expression network analysis (WGCNA) was conducted by the WGCNA package in the R statistical environment based on the expression profile data for each gene (Langfelder and Horvath, 2008). We did not perform merging the RNA-seq and microarray data, since the four transcriptome profiling datasets were collected from different broiler lines based on different selection criteria, and of different tissue origins (abdominal fat and preadipocytes). Instead, we constructed the network separately for each of the four data sets, and then compared the network modules obtained, to identify the common and line-specific modules associated with the adipogenesis trait, following the methods as described previously (Langfelder and Horvath, 2008). First, the raw gene expression data were pre-processed. We normalized and formatted the gene expression data into a data frame in the R statistical environment and filter out the null values of gene expression data. We performed sample clustering and gene expression analyses, respectively, and outliers (sample-wise or gene-/probe-wise) were detected and discarded. Second, soft threshold powers (β) were set first to fulfill the scale-free network assumption. In addition, we selected 5,000 genes with the highest connectivity for subsequent analysis based on the kRank, and calculated the weighted correlation values between genes to build the adjacency matrix. Third, the adjacency matrix was converted to a topological overlap matrix (TOM) to reduce noise and false correlation. Afterward, 1-TOM was calculated and treated as a biological important measure for network interconnectedness, and used as the distance measure for hierarchically clustered genes. Then, the dynamic tree cut algorithm was used to identify modules of co-expressed genes, and the module eigengenes (ME) were computed. Modules were merged based on the MEs, and labeled with different colors. Lastly, the specific modules were identified based on the module-trait relationship (pairwise Pearson’s correlation between ME and phenotypic trait values, and correlation coefficients >0.80). For abdominal fat tissue samples from the French lines, phenotypic traits were defined according to the bodyweights of the samples. As for the NEAUHLF tissue microarray samples, phenotypic traits included the bodyweights, the weights and percentages of abdominal fat tissues. For the preadipocyte microarray samples, we used directly their group properties, i.e., 1 and 0 for fat and lean chicken lines, respectively.

### Functional Analysis of Modules

We next performed the functional analysis of important hub genes and network modules. The hub genes were identified in each module based on the module membership (MM) values and the positional importance of genes in the co-expression network (Langfelder and Horvath, 2008). The detected hub genes of specific modules and their relationship were visualized in Cytoscape (version 3.6.1). Modules for each constructed co-expression network were compared, based on their molecular function and pathway importance. Furthermore, functional enrichment analysis was performed for the specific modules based on Gene ontology (GO) enrichment and Kyoto Encyclopedia of Genes and Genomes (KEGG) pathway analyses via DAVID (Database for Annotation Visualization and Integrated Discovery)^1^ (Huang et al., 2009). We then manually curated the identified significant pathways (*P* < 0.05). Based on the functional analysis results, we searched first common genes, and then common network sub-modules, to understand their relationship with adipogenesis.

### Functional Validation of Network Modules

Genes of important roles in modules related to chicken fat development were selected and studied by Real-time Quantitative PCR Detecting System (qPCR). The immortalized broiler cell lines ICP1 (Wang et al., 2017) were cultured and differentiated by oleic acid treatment *in vitro*. Total RNAs from cells undergoing differentiation were collected and extracted by the TRIZOL method. Genomic DNA was removed by the PrimeScript RT reagent Kit with gDNA Eraser. Using the SYBR Green method, the qPCR experiment system was set up on ABI7500 as follows: the total volume was 10 μL, including the forward and reverse primers (0.2 μL each), the cDNA template (1 μL), Fast Start Universal SYBR Green Master (ROX, 2×) (5 μL), and ddH_2_O (3.6 μL). The reaction conditions were as follows: 40 cycles of pre-denaturation at 95°C for 10 min, then denaturation at 95°C for 15 s, and lastly, extension at 60°C for 60 s. The TATA-box binding protein (*TBP*) was used as the reference gene, and data were analyzed by the 2^–ΔCt^ method (Livak and Schmittgen, 2001).

## Results

### Gene Co-expression Network Construction

To utilize effectively and accurately the public gene expression datasets, we began with standardized data preprocessing for each datasets. For one of the two datasets from the NEAUHLF chicken lines (the preadipocyte differentiation dataset), no outliers were detected, i.e., all 66 samples and 38,536 probes were kept (Supplementary Figure S1A); for the tissue expression dataset, two samples and probes were discarded (8 samples and 18,914 probes remained), respectively (Supplementary Figure S1B). As for the French datasets, none of the samples, but 2,649 probes, were discarded for the tissue RNA-seq dataset (24 samples and 15,286 transcripts remained) (Supplementary Figure S1C). And one sample, but no probe, was discarded for the tissue microarray (23 samples and 17,435 probes kept) (Supplementary Figure S1D).

**FIGURE 1:**
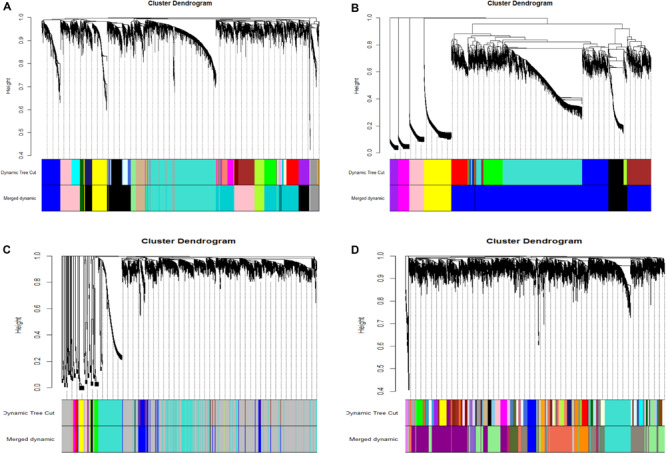
Clustering of co-expression modules. Upper panel: genes were clustered into different groups, and assigned to network modules after dynamic tree cut and merging analyses (Lower panel). **(A)** NEAUHLF: preadipocyte microarray. **(B)** NEAUHLF: adipose tissue microarray. **(C)** French: adipose tissue RNA-seq. **(D)** French: adipose tissue microarray.

After the model comparison (the scale free topology model vs. the fitted model) and by considering *R*^2^ >0.8, we found soft thresholds (β = 9, 12, 12, 10) for the preadipocyte and tissue microarrays of NEAUHLFF, and the RNA-seq and tissue microarray datasets of French lines, respectively (Supplementary Figure S2). Then, for the construction of co-expression networks, we selected 5,000 genes with the most expression variation, and transformed the adjacency matrix into topology matrix. Dynamic tree cut algorithm was used to merge all modules (correlation coefficients >0.75, and lowest number of genes in the module set at 30) (Figure 1). For the four datasets (the preadipocyte and tissue microarrays of NEAUHLF lines, and the tissue RNA-seq and microarray datasets of French lines), 13, 6, 12, and 17 modules were detected, respectively (Figures 1A–D). And specifically, modules containing the largest and least number of genes were Turquoise (1,173) and Darkgray (42) (Figure 1A), Blue (3,457) and Purple (163) (Figure 1B), Turquoise (1,173) and Tan (33) (Figure 1C), Lightgreen (838) and Mediumorchid (31) (Figure 1D), respectively.

### Identification of Modules of Interests

**FIGURE 2:**
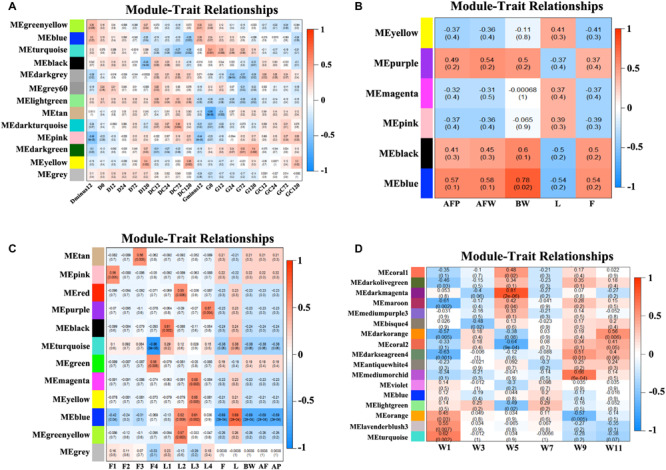
Module-trait heatmaps. Gene modules and trait relationships established by correlation analyses. **(A)** NEAUHLF: preadipocyte microarray. **(B)** NEAUHLF: adipose tissue microarray. **(C)** French: adipose tissue RNA-seq. **(D)** French: adipose tissue microarray.

We obtained the module-trait heatmaps by calculating the correlation coefficients between modules and traits for the four datasets, respectively (Figure 2). First, for the NEAUHLF preadipocyte dataset, the Turquoise module was highly positively correlated with the preadipocyte differentiation group after oleate treatment for both fat and lean lines, but negatively correlated with the control groups (Figure 2A). Moreover, the Turquoise module was significantly positively correlated with the induced differentiation group of the fat line, but the early differentiation group showed stronger correlation than the late stage group. As for the lean line, the Turquoise group was significantly negatively correlated with the control group, but showing stronger correlation for the late stage than the early stage. So, genes in the Turquoise module could promote the differentiation, but inhibit the proliferation of preadipocytes. The Lightgreen module, in contrast, was positively correlated with both the induced differentiation and the control groups for the lean chicken line, but negatively correlated with all treatment and control groups of the fat line, indicating that the Lightgreen module could inhibit preadipocyte proliferation, and enhance lipid catabolism. Moreover, we found that the Darkgreen module was positively correlated with the induced differentiation group of the fat line, but negatively correlated with the control group of the lean line. The degree of correlation appeared in a progressively increasing or decreasing manner for the fat and lean lines, respectively. Thus, genes in the Darkgreen module could enhance the preadipocyte differentiation, and promote adipogenesis.

As for the NEAUHLF tissue microarray dataset, we didn’t find any strong correlation with the traits for most of the modules (Figure 2B). Only the Blue module correlated strongly with the bodyweights, and relatively strongly with both the abdominal fat rate and weight, too. Whereas for the French tissue RNA-seq dataset, the Blue module correlated positively with the lean line, but negatively with the fat line (all most significantly), respectively. Genes with negative effects on fat deposition might be contained in the Blue module (Figure 2C).

For the French tissue microarray dataset (Figure 2D), the Turquoise and Orange modules were positively correlated with the bodyweight at 1 week of age, but negatively correlated with those at 11 and 9 weeks of age, respectively. In contrast, the Darkseagreen4 and Darkorange modules showed the opposite trend, correlated negatively with the bodyweight at 1 week of age, but positively with bodyweights at 9 and 11 weeks of ages (all most significantly), respectively. Genes in these modules could have potential effects on fat deposition. Thus, our module-trait correlation analyses pinpoint network modules potentially underlying adipogenesis or the growth and development of chicken adipose tissue.

### Hub Genes in Important Sub-Modules

Hub gene occupies central position, and is of vital function in the gene network, which is determined by their connectedness with other genes in the network. Our previous analyses helped find nine functional modules of importance to adipose tissue growth and development, after we built the network based on the threshold of correlation coefficients (>0.85) (Table 1). Then, we selected 3–6 hub genes from each module. Functional exploration revealed that these genes were related to adipogenesis, cell cycle, inflammation and protein synthesis. In addition, we extracted and plotted the gene expression values of hub genes, which showed that different hub genes in the same module were of similar expression patterns (Supplementary Figure S3).

**TABLE 1:** Hub genes in modules identified to be related to chicken fat development.

**Dataset**	**Module (threshold)**	**Hub genes**
NEAUHLF preadipocyte	C-Turquoise (0.96)	BRD1, DST, CCDC88A, SBNO1, VCPIP1, ROCK2
	Lightgreen (0.88)	ASCC1, ZDHHC20, CCNG2, RRM2B, C10H15ORF40, ORAOV1
	Darkgreen (0.88)	PTN, CMTM4, CORO2A, IGF2BP2
NEAUHLF tissue	T-Blue (0.987)	WDR1, METAP2, XPOT, PREX2
French tissue RNA-seq	F-Blue (0.92)	SH3GLB1, FABP5, PIK3R2, AGRN, ADAMTS7, RPTPF
French tissue microarray	T-Turquoise (0.95)	PLCL2, NT5M, TJP1, ACTR1A
	Orange (0.87)	PGRMC1, FADS2, CAV2
	Darkseagreen4 (0.9)	KCNV1, PIK3CD, PAH, FARSB, SH3BP4
	Darkorange (0.9)	CCNL2, THSD7B, PTCD3

### Common Modules Identified by Network Comparison

**FIGURE 3:**
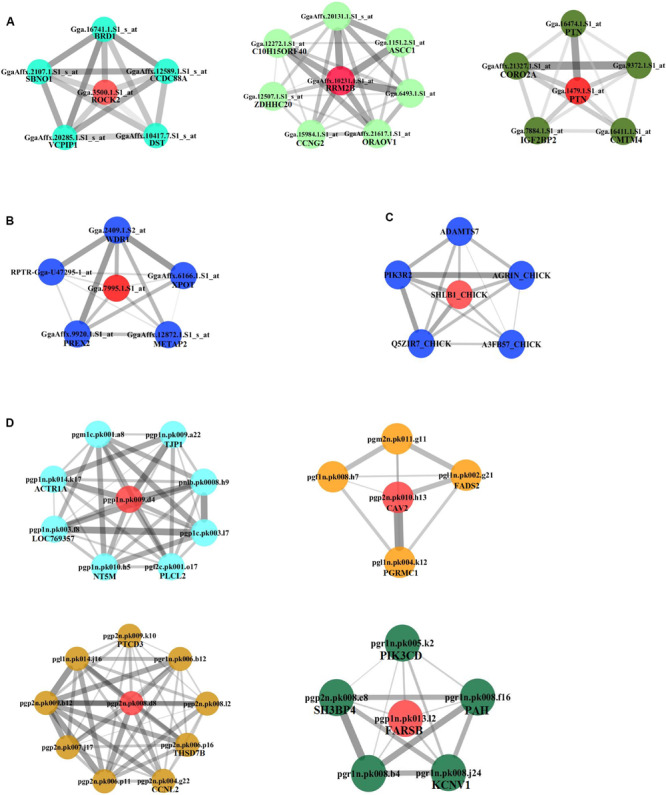
Subnetworks constructed for hub genes. Direct gene neighbors of the hub genes were extracted for building subnetworks. Four subnetworks were built. **(A)** NEAUHLF: preadipocyte microarray. **(B)** NEAUHLF: adipose tissue microarray. **(C)** French: adipose tissue RNA-seq. **(D)** French: adipose tissue microarray.

We continued to build the gene co-expression sub-networks around the discovered hub genes, in an attempt to show the importance and implication of these genes on adipogenesis. For the four datasets, 3, 1, 1, and 4 sub-networks were found, respectively (Figure 3). After functional enrichment analyses and literature retrieval on central hub genes (such as *ROCK2*, *RRM2B*, *PTN*, *CAV2*, *FARSB*, *SH3GLB1*, *FABP5*, and *FADS2*), interestingly, we found great similarity for biological processes and molecular pathways (Supplementary Table S1). For many modules constructed, development and regulation of adipose tissue and metabolic pathways were found to be enriched, such as lipid storage, lipid modification, negative regulation of lipid synthesis, fatty acid metabolism, PPAR and insulin signaling pathways. In addition, after GO analysis, modules were found to be enriched in a variety of pathways, such as protein hydrolysis, transport and localization, cytoskeleton, apoptosis and programmed cell death. KEGG pathway analysis also identified two pathways, focal adhesion and proteasome metabolism (Supplementary Table S1).

**FIGURE 4:**
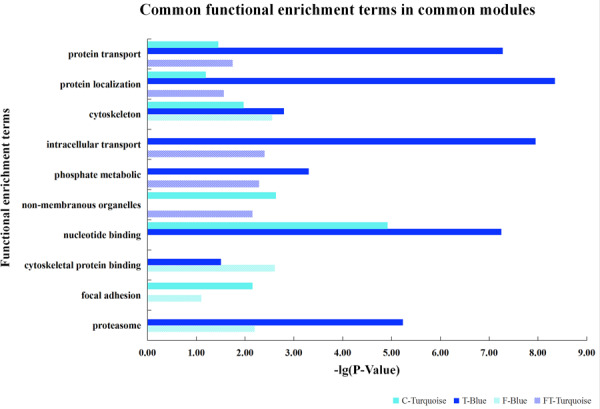
Common gene modules discovered for different chicken lines.

Furthermore, based on the functional consistency between identified important modules, common gene modules were found (e.g., Turquoise, Blue, Blue, and Turquoise modules for the four datasets, the NEAUHLF preadipocyte and tissue microarrays, and the French tissue RNA-seq and microarray datasets, respectively, even though labeled with different color codes) (Figure 4). These common modules were enriched in the establishment of cytoskeleton, and protein metabolic transport and localization. Additional analyses found many modules with similar functional enrichment, such as cell apoptosis and programmed cell death (Supplementary Table S2). In contrast, modules specific to chicken lines and having their specific functionality to chicken adipogenesis were also found. Thus, in chicken lines divergently selected for abdominal fat contents, though of different genetic backgrounds, common gene modules with similar functionality were detected, and could potentially play conserved roles in the growth and development of adipose tissues.

### Validation of the Lysosome Module

**FIGURE 5:**
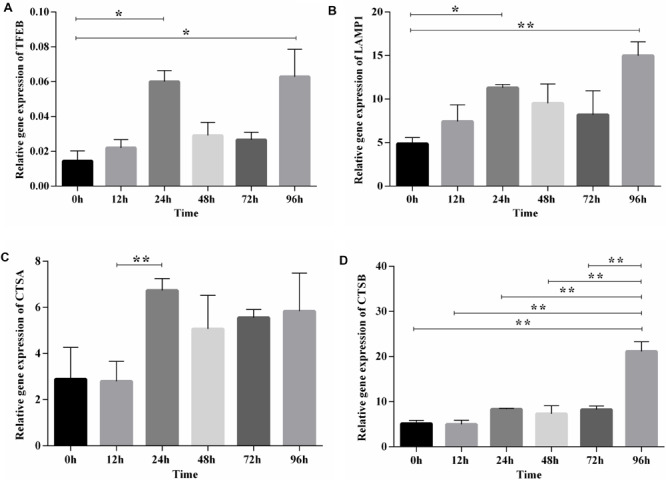
The expression and significance test of TFEB and its downstream genes during induced differentiation of ICP1 preadipocyte line. **(A)** TFEB. **(B)** LAMP1. **(C)** CTSA. **(D)** CTSB. Mean significance at *P < 0.05 and **P < 0.01 levels.

After functional enrichment analysis, the lysosome pathway was among those pathways discovered to be potentially important for chicken adipose tissue growth and development. Lysosome has a vital role in lipid metabolism, and we and others showed previously that the lysosome pathway is fundamental to chicken adipogenesis (Thelen and Zoncu, 2017; Data not shown). Here we selected *TFEB* (the master regulator of lysosome biogenesis and also involved in lipid metabolism), and also its downstream genes (*LAMP1*, *CTSA*, *CTSB*), to examine their expression patterns during preadipocyte differentiation in the preadipocyte cell line (ICP1) (Supplementary Figure S4). The expression dynamics of *TFEB* and its downstream genes exhibited a similar pattern, and highly correlated with each other. Furthermore, obvious changes along with the differentiation of preadipocytes in broilers could be seen, since significant differences existed between these genes at different time points (Figure 5). Thus, the lysosome pathway may play a regulatory role in the differentiation of chicken preadipocytes.

## Discussion

In the current study, gene co-expression networks were built for two different chicken lines both under divergent selection for abdominal fat content, and common network modules with similar functionality were discovered to be of potential importance for adiposity. Our findings could provide novel insights into the genetic basis of complex traits, and help understand the outcomes of intensive breeding practices in modern animal production systems.

The recently developed high-throughput RNA-seq technology propels the study on the detection of differentially expressed genes by statistical modeling and analysis on transcriptome profiling data. However, the co-linearity and confounding factors embedded within the gene expression data (usually small number of samples, but large number of parameters, i.e., the n << p problem) could not be discerned by these normal statistical methods (Barabási and Oltvai, 2004; Ghaemi et al., 2019). To cope with the high-dimensional data analysis question induced by large number of genes, methods for the construction of gene networks were developed, such as the gene co-expression network (Langfelder and Horvath, 2008; Banf and Rhee, 2017; Mangul et al., 2019), and have been effectively used in identifying the topological relatedness and interaction of genes in the biological systems of interests (Lee et al., 2016; Emilsson et al., 2018). Nevertheless, the spatio-temporal and heterogeneous characteristics of the transcriptome data require the deployment of novel statistical methods (e.g., machine learning), to effectively solve the statistical and modeling issues related to network dynamics and heterogeneity (Camacho et al., 2018).

We identified network modules significantly associated with fat deposition in chicken lines with different genetic backgrounds, but all under divergent selection for the same trait of interest, abdominal fat content (Guo et al., 2011; Resnyk et al., 2013, 2017). Previously, after differential expression analyses on the same datasets, genes involved in fatty acid metabolism and PPAR signaling pathways were found, such as *PPARG*, and its direct targets, *SCD*, *ACSL1*, and *DGAT2* (Guo et al., 2011; Resnyk et al., 2013, 2017), which potentially explained the underlying differences of fat deposition in these chicken lines. However, with gene co-expression network analysis, we found network modules containing interesting genes in molecular pathways (fatty acid metabolism, PPAR and insulin, cytoskeleton, and protein synthesis, etc.) fundamental to adipose tissue growth and development, such as *FABP5*, *FADS2*. The insulin pathway was found to be vital to fatty acid content composition in cattle (de Oliveira et al., 2019), and *FABP5* and *FADS2* are well-known regulatory genes on fatty acid metabolism (Li et al., 2019; Xing et al., 2019). Cytoskeleton has to reorganize with adipose tissue remodeling and expansion, and recently cytoskeletal transgelin 2 was proved to be associated with preadipocyte proliferation and differentiation (Ortega et al., 2019). Protein synthesis is regulated mainly by the master growth regulator *mTOR* (Thoreen et al., 2012), which plays a vital role in lipid metabolism (Caron et al., 2015). Further expression pattern analysis showed that these genes in the same module did have similar expression levels and dynamics, which could be transcriptionally regulated by a common set of transcription factors (Gachon et al., 2018; Klemm et al., 2019). These discovered genes and signaling pathways seem to be common and have important conserved functions for adipogenesis, which could be partially due to that divergent selection put more forces on gene modules of common fundamental roles in adipogenesis, and were picked up by our network module comparison analysis. Further detailed investigation on the transcriptional regulation of genes in these identified network modules could render novel insights into the underlying molecular regulatory mechanisms of these chicken lines.

Integrated genomics and network science methods are widely employed in systems biology, to fully utilize the large volume of genomics and biological data (Lee et al., 2016; Emilsson et al., 2018; Ghaemi et al., 2019). We herein detected common molecular network and functional pathways involved in abdominal fat deposition after network construction and comparison between different chicken lines. It’s a common phenomenon that similar traits of animals in different populations could be under convergent selection, i.e., different genes but pathways of similar molecular functions are selected by evolutionary forces or artificial selective pressure, such as convergent selection signatures in sheep and goat (Alberto et al., 2018), and the frizzle phenotype in chickens (Dong et al., 2018; Guo et al., 2018). Here, our network analysis also showed that, though different chicken lines were divergently selected for abdominal fat content, network modules of similar molecular functionality were detected. These results could provide further insights on the genetics of complex trait, including human diseases.

## Conclusion

Gene co-expression networks were constructed for different chicken lines under divergent selection for adiposity. Common sub-modules of similar functionality for chicken fat deposition were identified after gene functional enrichment analysis and network comparison. Our findings indicate that even in different chicken lines, common molecular pathways could be underlying the growth and development of adipose tissue.

## Data Availability Statement

Publicly available datasets were analyzed in this study. This data can be found here: GSE51330, GSE8010, GSE42980, and GSE37585.

## Ethics Statement

The animal study was reviewed and approved by Laboratory Animal Management Committee of Yangtze University.

## Author Contributions

ZG analyzed data and wrote the manuscript. ZG and RD performed the experiment. XZ, YW, and YC participated in the experiment. Z-QD and C-XY designed the experiment, analyzed data, and wrote the manuscript.

## Conflict of Interest

The authors declare that the research was conducted in the absence of any commercial or financial relationships that could be construed as a potential conflict of interest.

## Footnotes

1 https://david-d.ncifcrf.gov/tools.jsp

## Supplementary Material

FIGURE S1 Cluster analyses of samples and gene expression data to detect outliers. No outliers found for **(A)** (NEAUHLF: preadipocyte microarray), whereas two samples and two genes were discarded for **(B)** (NEAUHLF: adipose tissue microarray). As for the French datasets, 2,649 probes were discarded for the adipose tissue RNA-seq dataset **(C)** and one sample outlier was discarded for the adipose tissue microarray **(D)**.

FIGURE S2 Identification of soft thresholds. The soft thresholds β = 9, 12, 12, 10 for preadipocyte **(A)** and adipose tissue **(B)** microarrays of NEAUHLFF, and adipose tissue RNA-seq **(C)** and microarray **(D)** of French lines.

FIGURE S3 Gene expression profiles of hub genes in the important modules. Consistent and highly correlated gene expression patterns were identified for hub genes in the network sub-modules, for the four datasets, respectively.

FIGURE S4 Culture and induced differentiation of chicken preadipocyte cell line. Oil red oxygen staining **(A)** and gene expression trend of differentiation markers of adipocytes in broilers **(B)**.

TABLE S1 Enrichment analysis of modules.

TABLE S2 Summary of hub genes in modules related to fat development.
