# Supplementary material for: Common Gene Modules Identified for Chicken Adiposity by Network Construction and Comparison
Source: Front Genet. 2020 May 29;11:537. doi: 10.3389/fgene.2020.00537 (PMC7272656; doi:10.3389/fgene.2020.00537)
Supplement: FIGURE S1 — Cluster analyses of samples and gene expression data to detect outliers. No outliers found for (A) (NEAUHLF: preadipocyte microarray), whereas two samples and two genes were discarded for (B) (NEAUHLF: adipose tissue microarray). As for the French datasets, 2,649 probes were discarded for the adipose tissue RNA-seq dataset (C) and one sample outlier was discarded for the adipose tissue microarray (D). [file Data_Sheet_1.docx]

**Supplementary Figure S1.**


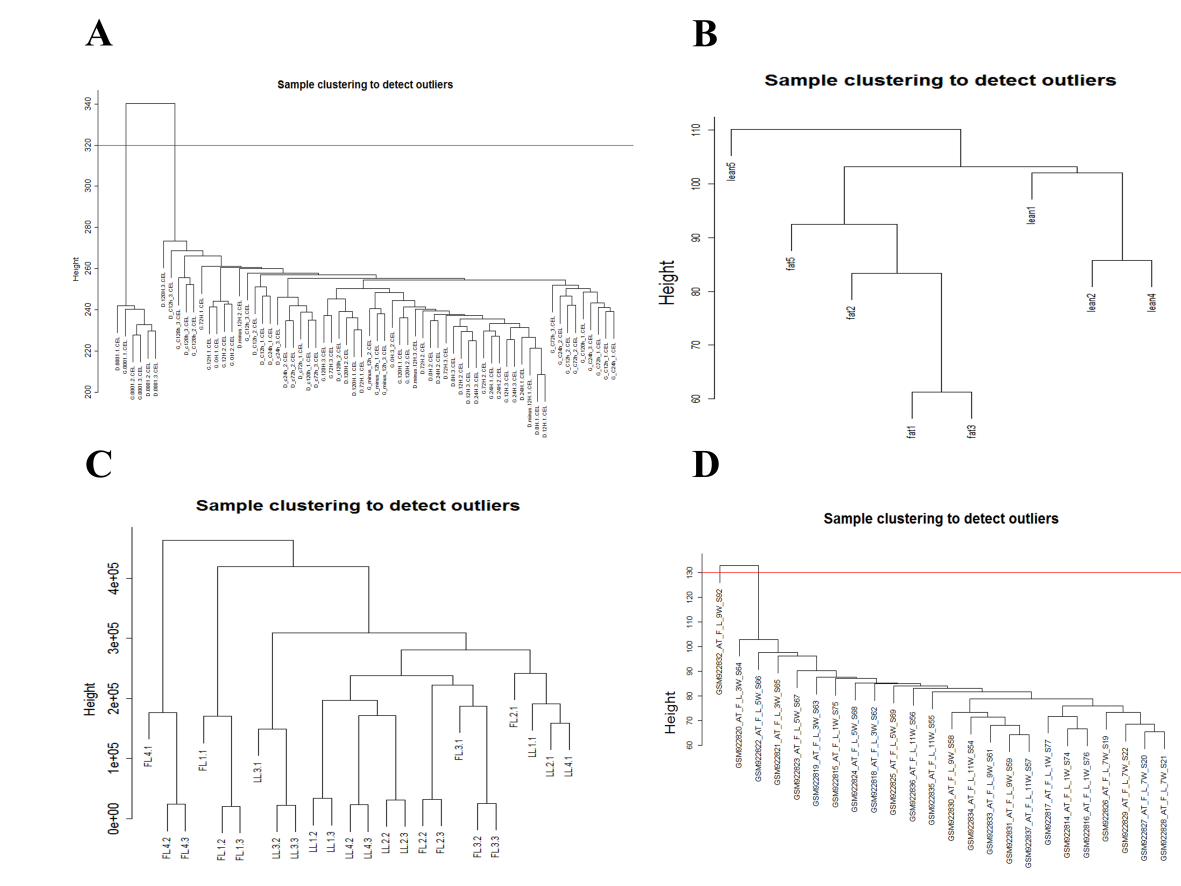


**Supplementary Figure S2.**


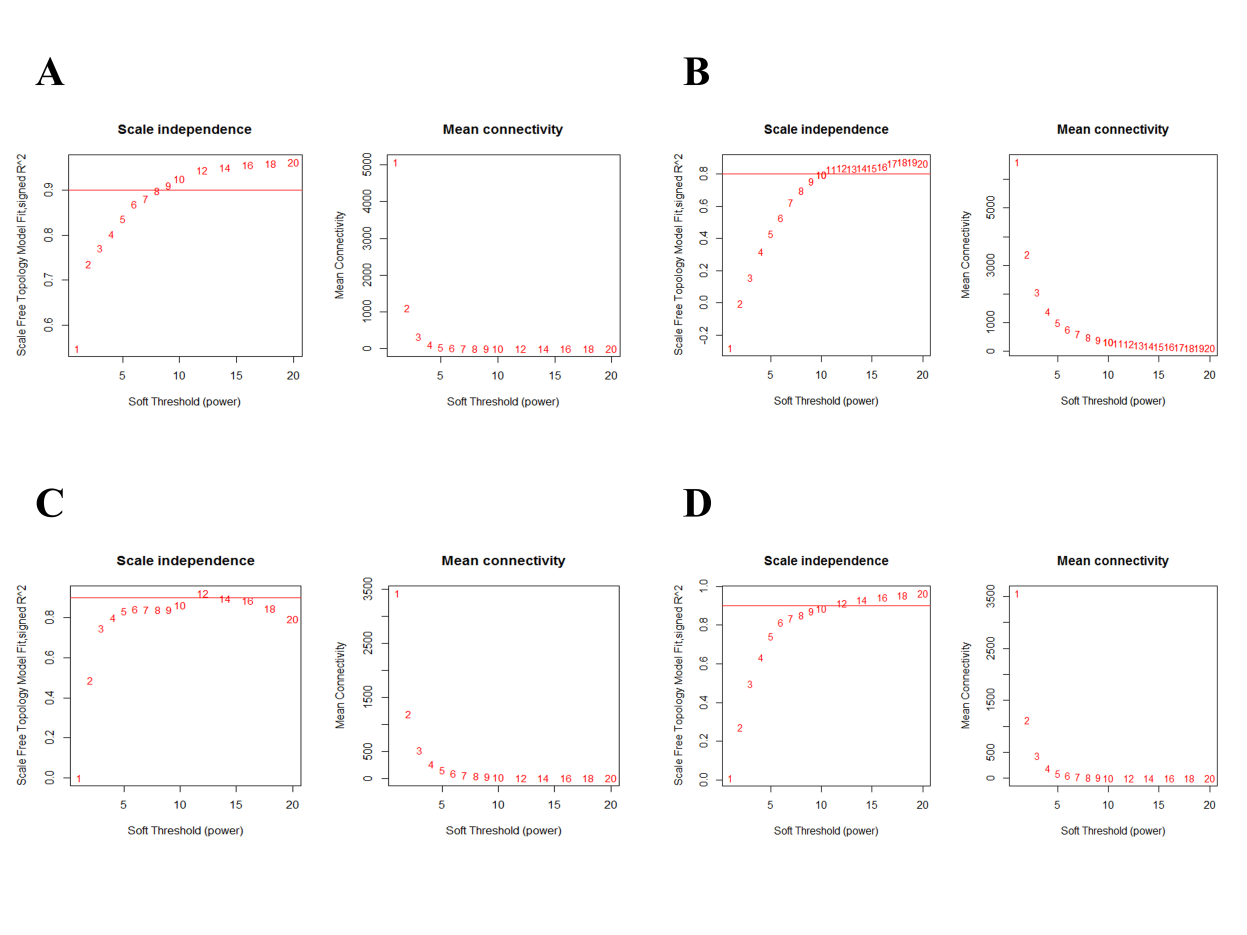


**Supplementary Figure S3**

**
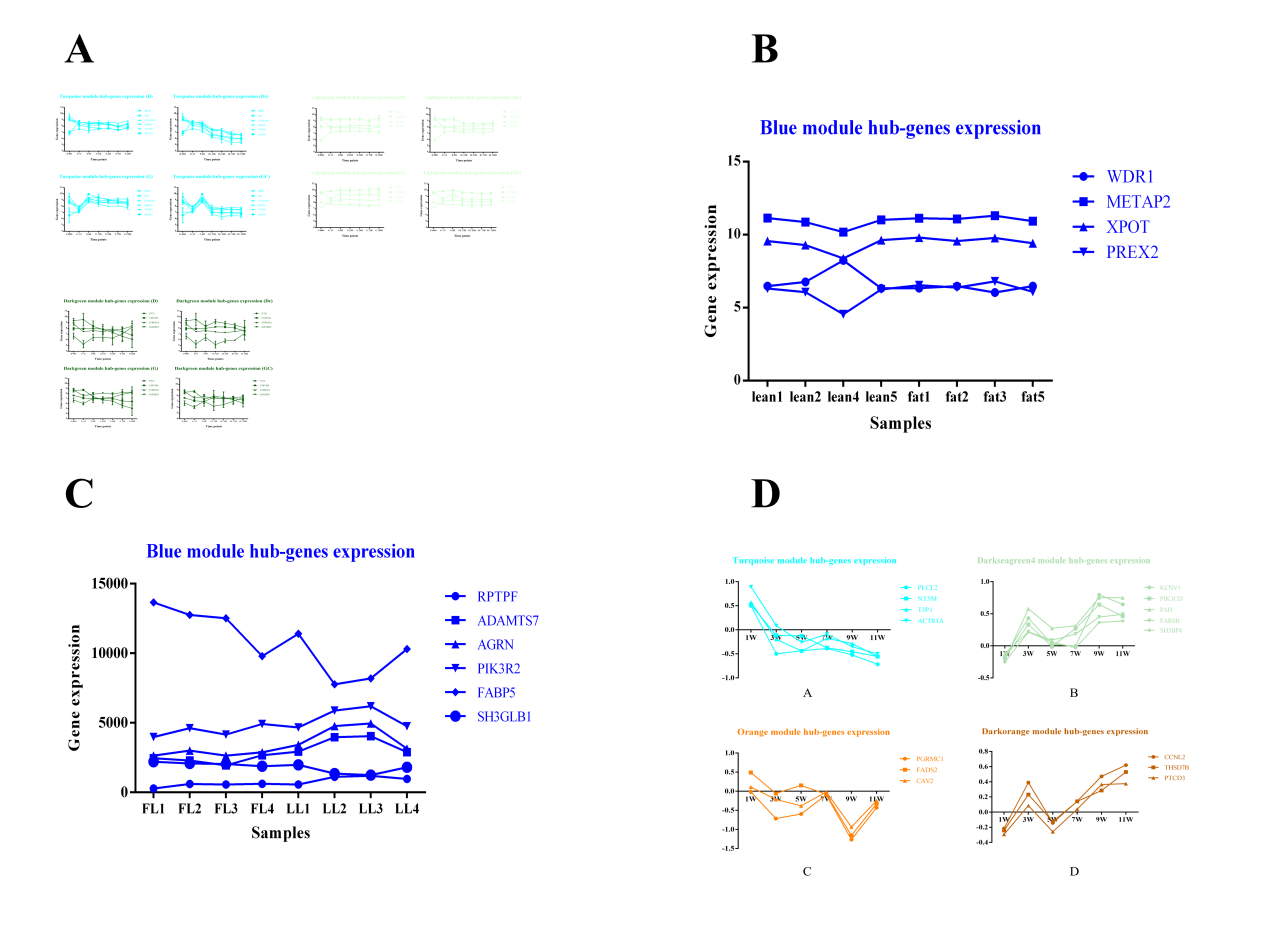
**

**Supplementary Figure S4**

**
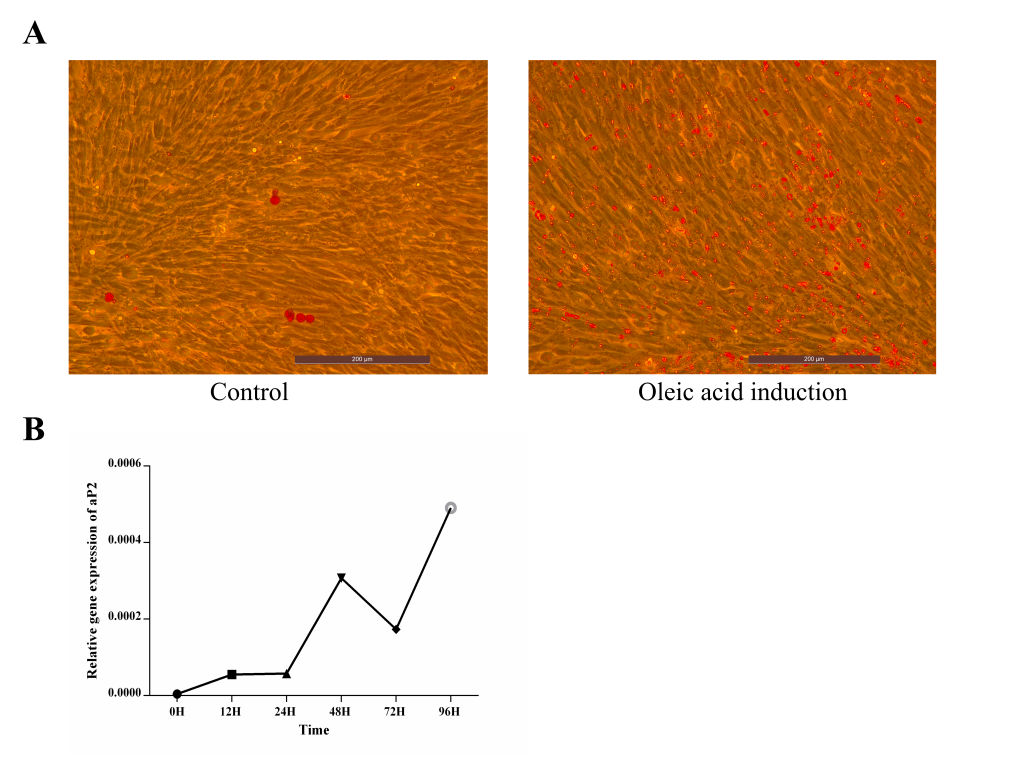
**
