# Supplementary material for: Common Gene Modules Identified for Chicken Adiposity by Network Construction and Comparison
Source: Front Genet. 2020 May 29;11:537. doi: 10.3389/fgene.2020.00537 (PMC7272656; doi:10.3389/fgene.2020.00537)
Supplement: TABLE S1 — Enrichment analysis of modules. [file Data_Sheet_2.docx]

**Supplementary Table S1. Enrichment analysis of modules**

| Module | GO_BP | GO_CC | GO_MF | KEGG |
| --- | --- | --- | --- | --- |
| C-Turquoise | Intracellular signaling cascade; mRNA processing; mRNA metabolic process; Protein catabolic process; Cytoskeleton organization; Cell cycle; Establishment of protein localization | Non-membrane-bounded Organelle; Cytoskeleton; Chromosome; Vesicle | Nucleotide binding; DNA binding; ATP binding; Ribonucleotide binding; Phospholipid binding | Insulin signaling pathway; Dorso-ventral axis formation; Focal adhesion; ErbB signaling pathway; GnRH signaling pathway; Vascular smooth muscle contraction |
| Lightgreen | Cell redox homeostasis; Apoptosis; Histone modification; Programmed cell death; Covalent chromatin modification; Histone deacetylation | Mitochondrion; Pre ribosome; Ribonucleoprotein complex | Electron carrier activity; Protein disulfide oxidoreductase activity; Disulfide oxidoreductase activity | p53 signaling pathway |
| Darkgreen |  |  | Zinc ion binding |  |
| T-Blue | Regulation of lipid storage; Protein transport; Establishment of protein localization; Protein catabolic process; Proteolysis; Regulation of cytoskeleton organization; Phosphate metabolic process | Lysosome; Cytosol; Mitochondrion; Proteasome complex | Nucleotide binding; RNA binding; Protein transporter activity; Phosphatase activity; Cytoskeletal protein binding | Focal adhesion; Lysosome; PPAR signaling pathway; Wnt signaling pathway; Proteasome; Oxidative phosphorylation; Regulation of actin cytoskeleton |
| F-Blue | Epithelial cell differentiation; Epithelium development; Regulation of organelle organization; Positive regulation of protein kinase cascade; Regulation of actin cytoskeleton organization; Regulation of cellular component biogenesis; Cellular component morphogenesis | Extracellular space; Extracellular region part; Fibrinogen complex; Cell cortex | Protein binding; Cytoskeletal protein binding; Translation factor activity, nucleic acid binding; Myosin binding; Enzyme inhibitor activity | Proteasome; Tight junction |
| FT-Turquoise | Negative regulation of lipid biosynthetic process; Intracellular transport; Regulation of phosphate metabolic process; Negative regulation of neuron apoptosis; Regulation of cell morphogenesis; Intracellular protein transport; Chemical homeostasis; Cell growth; ATP metabolic process; Cellular protein localization; Regulation of cell death | Non-membrane-bounded Organelle; Dendrite; Cytoskeleton; Cell surface | Structural molecule activity; Calmodulin binding; ATPase activity, coupled to transmembrane Movement of ions | Arginine and proline metabolism |
| Orange | Fatty acid metabolic process; Fatty acid biosynthetic process; Mbryonic development ending in birth or egg hatching |  | Cofactor binding | Alpha-Linolenic acid metabolism |
| Darkseagreen4 | Second-messenger-mediated signaling; Lipid modification; Lipid phosphorylation; Regulation of osteoclast differentiation; Positive regulation of cell differentiation | Cell fraction; Membrane fraction; Insoluble fraction | Inositol or phosphatidylinositol kinase activity |  |
| Darkorange | Protein folding; Membrane protein proteolysis; Membrane protein ectodomain proteolysis | Plasma membrane; Membrane fraction; Insoluble fraction; Cytosol; Cell fraction | Unfolded protein binding; Oxidoreductase activity |  |

**Supplementary Table S2. Summary of hub genes in modules related to fat development**

| Category | Term | Module |
| --- | --- | --- |
| GO_BP | Many terms riched in different modules are all relative to adipose: lipid storage, lipid modification, negative regulation of lipid synthesis, fatty acid metabolism | T-Blue; Darkseagreen4; Orange; FT-Turquoise |
| GO_BP | Protein Metabolism, Hydrolysis and Transport | C-Turquoise; T-Blue; FT-Turquoise; Darkseagreen4 |
| GO_BP | protein localization | C-Turquoise; T-Blue; FT-Turquoise; Darkseagreen4 |
| GO_BP | Cytoskeleton | C-Turquoise; T-Blue; F-Blue |
| GO_BP | Apoptosis | Lightgreen; FT-Turquoise |
| GO_BP | Programmed cell death | Lightgreen; FT-Turquoise |
| GO_BP | intracellular transport | T-Blue; FT-Turquoise |
| GO_BP | Phosphate metabolic | T-Blue; FT-Turquoise |
| GO_BP | Morphogenesis of cells | T-Blue; FT-Turquoise |
| GO_CC | non-membranous organelles | C-Turquoise; FT-Turquoise |
| GO_CC | Proteasome | Lightgreen; T-Blue |
| GO_CC | Cytosol | T-Blue; Darkorange |
| GO_CC | mitochondrion | Lightgreen; T-Blue |
| GO_MF | nucleotide binding | C-Turquoise; T-Blue |
| GO_MF | cytoskeletal protein binding | T-Blue; F-Blue |
| KEGG | Focal adhesion | C-Turquoise; F-Blue |
| KEGG | Proteasome | T-Blue; F-Blue |
